# Moderate Static Magnetic Field (6 mT)-Induced Lipid Rafts Rearrangement Increases Silver NPs Uptake in Human Lymphocytes

**DOI:** 10.3390/molecules25061398

**Published:** 2020-03-19

**Authors:** Cristian Vergallo, Elisa Panzarini, Bernardetta Anna Tenuzzo, Stefania Mariano, Ada Maria Tata, Luciana Dini

**Affiliations:** 1 Department of Biological and Environmental Science and Technology, University of Salento, 73100 Lecce, Italy; cristian.vergallo@unisalento.it (C.V.); elisa.panzarini@unisalento.it (E.P.); bernardetta.tenuzzo@unisalento.it (B.A.T.); stefania.mariano@unisalento.it (S.M.); 2 Department of Biology and Biotechnology “Charles Darwin”, Sapienza University of Rome, 00185 Rome, Italy; adamaria.tata@uniroma1.it; 3 CNR Nanotec, 73100 Lecce, Italy

**Keywords:** static magnetic field (SMF), peripheral blood lymphocytes (PBLs), silver nanoparticles (AgNPs) uptake, disialoganglioside GD3, cholesterol, ATP-binding cassette transporter A1 (ABCA1), lipid rafts

## Abstract

One of the most relevant drawbacks in medicine is the ability of drugs and/or imaging agents to reach cells. Nanotechnology opened new horizons in drug delivery, and silver nanoparticles (AgNPs) represent a promising delivery vehicle for their adjustable size and shape, high-density surface ligand attachment, etc. AgNPs cellular uptake involves different endocytosis mechanisms, including lipid raft-mediated endocytosis. Since static magnetic fields (SMFs) exposure induces plasma membrane perturbation, including the rearrangement of lipid rafts, we investigated whether SMF could increase the amount of AgNPs able to pass the peripheral blood lymphocytes (PBLs) plasma membrane. To this purpose, the effect of 6-mT SMF exposure on the redistribution of two main lipid raft components (i.e., disialoganglioside GD3, cholesterol) and on AgNPs uptake efficiency was investigated. Results showed that 6 mT SMF: (i) induces a time-dependent GD3 and cholesterol redistribution in plasma membrane lipid rafts and modulates gene expression of ATP-binding cassette transporter A1 (ABCA1), (ii) increases reactive oxygen species (ROS) production and lipid peroxidation, (iii) does not induce cell death and (iv) induces lipid rafts rearrangement, that, in turn, favors the uptake of AgNPs. Thus, it derives that SMF exposure could be exploited to enhance the internalization of NPs-loaded therapeutic or diagnostic molecules.

## 1. Introduction

The improvement in designing nanosized materials (NMs) to be used as a delivery system is an important goal in nanomedicine. Silver nanoparticles (AgNPs) are receiving significant attention in improving timed/controlled intracellular drug and imaging agent deliveries [1]. Moreover, the optical properties of AgNPs could be used in cancer therapy based on the application of light, such as photodynamic therapy [2]. To better optimize AgNPs biomedical applications, two main aspects are investigated, i.e., safer synthesis methods and deeper knowledge of NPs cellular uptake mechanisms [3]. Literature data suggest that size and surface-coating of AgNPs may dictate cell uptake [4,5,6] and concord that the internalization pathways are clathrin- [4] and lipid rafts- [7] mediated endocytosis. In previous papers, we demonstrated that AgNPs synthetized by using β-d-glucose (AgNPs-G) as a reducing agent to prevent dissolution of Ag^+^ enter Hela cells [8] and human peripheral blood lymphocytes (PBLs) [9] in an amount- and incubation time-dependent manner. In the present study, we attempt to improve the uptake of AgNPs-G by PBLs via modulation of plasma membrane fluidity. We chose to use a static magnetic field (SMF) exposure to rearrange the lipid rafts, as most of evidence-based findings suggest that the plasma membrane is the elective target of MFs due to its anisotropic and diamagnetic properties.

MFs of moderate magnetic induction (10^−3^ ≤ B < 1 T) are able to influence biological systems by interacting with lipids [10], proteins [10,11,12,13] and glycans [11,14,15,16] of plasma membrane in exposure time- and cell type-dependent extent. In turn, these plasma membrane changes may trigger a cascade of cell responses, e.g., cytoskeletal rearrangements, redistribution of receptors along cell surface, ion flux alteration, etc., and could also affect uptake processes, including NPs internalization.

Among target molecules of SMF exposure, monosialoganglioside GM3 and its desialylated derivative GD3 are pivotal, as they are coupled with sphingomyelin and cholesterol in dynamic microdomains of the plasma membrane, called lipid rafts. The lipid raft domains of the plasma membrane are small, highly dynamic and transient plasma membrane entities enriched in saturated phospholipids, sphingolipids, glycolipids, cholesterol, lipoproteins and glycosylphosphatidylinositol (GPI)-anchored proteins. It is widely demonstrated that lipid rafts: (i) regulate plasma membrane transport and signaling, due to their high affinity for ion channels and signal transduction proteins; (ii) play a role in cell adhesion and cell migration, by interacting with integrins and cytoskeleton; (iii) play a role in host-pathogen interactions and colonization of bacteria, viruses and prions and (iv) participate in the protein and lipid sorting at the trans-Golgi network and in the apical delivery in polarized cells [17]. Furthermore, raft-like domains, whose role remains still unclear, are also identified in the membranes of different subcellular organelles [18]. Interestingly, SMF exposure affects exposure and localization of monosialoganglioside GM3 and its desialylated derivative GD3. Thus, the study itself of these dynamic microdomains of plasma membrane is of great interest, and it becomes more interesting in view of a possible strategy to increase drug-loaded NPs entry into the cells.

To this purpose, in order to exploit the influence of 6-mT SMFs on plasma membrane lipid rafts contributing to the possible AgNPs increased uptake, we performed an in vitro study by comparing two different culture conditions of human PBLs, i.e., in the presence or in the absence of 6-mT SMFs, to study the effect of lipid raft rearrangement on AgNPs-G uptake. The presence and distribution of GD3, cholesterol, ATP-binding cassette transporter A1 (ABCA1) gene expression levels, reactive oxygen species (ROS) production, plasma membrane lipid peroxidation and PBLs viability was evaluated. Our results encourage further investigation on the possibility that SMF exposure could be exploited to enhance the internalization of NPs-loaded therapeutic or diagnostic molecules.

## 2. Results

### 2.1. Effects of 6 mT SMF on PBLs Viability

Cultures of isolated PBLs show a progressive decrement due to spontaneous cell death unless they are supplemented with a mitogen, like PHA, able to transform large population of lymphocytes in mitotically active cells, and thus, they can be maintained in culture up to 1 week by preventing cell death (Figure S1). The exposure to 6 mT SMF of PHA treated PBLs does not influence cell viability of PBLs (Figure S2).

Control PBLs during 48 h of culture showed smooth cell surface, rounded cell shape and nucleus slightly off-centered, high nucleus/cytoplasm ratio. Only after 72 h of culture, due to massive blebs-like protrusions, a wrinkled cell surface was observed. A morphology comparable to not-exposed PBLs was displayed within 24 h of SMF exposure. At the longest time of SMF exposure, the cell shape became elongated with blebs. A time-dependent morphological modification typical of apoptosis (i.e., elongated cell shape, pyknotic nucleus, condensed and/or fragmented chromatin and nucleus, and extensive plasma membrane blebbing) was found in cycloheximide (CHX)-treated cells. Cells simultaneously CHX-treated and SMF-exposed showed less typical apoptotic features when compared to CHX treatment only.

**Figure 1:**
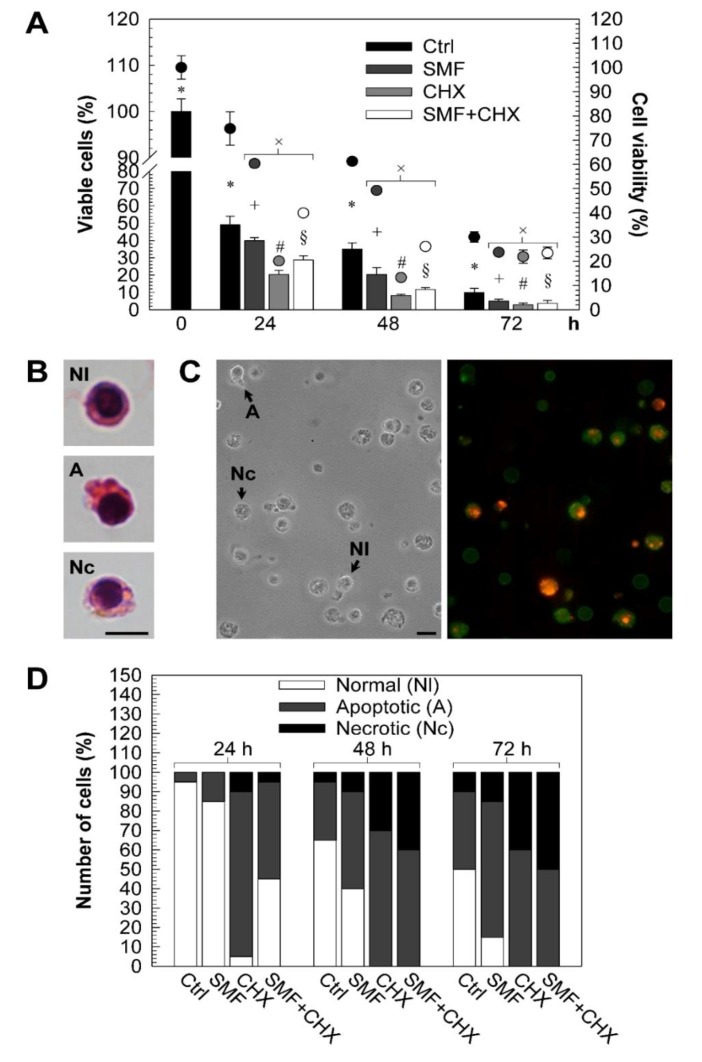
Cell viability. (**A**): Percentage of viable peripheral blood lymphocytes (PBLs) following the different treatments by trypan blue dye exclusion assay (histograms) or MTT assay (dots). All values referred to the value of control PBLs at 0 h, taken as 100%. Each error bar represents the SE of five independent experiments, performed in duplicate. × indicates significant values *vs* control (*p* < 0.05); +, #, § (*p* < 0.0167) and * (*p* < 0.0083) indicate significant values *vs* those indicated with the same symbol (**B**–**C**): Representative light microscopy (LM) micrographs of normal (Nl), apoptotic (A) and necrotic (Nc) PBLs stained with H&E, after fixation with 4% formaldehyde (B) or labeled with annexin V-FITC/propidium iodide (C). (**D**): Percentage of annexin V-FITC/propidium iodide labeled normal, apoptotic and necrotic PBLs following different treatments scored at LM. For each experiment, at least 500 cells were counted. The SE refers to five independent experiments each performed in duplicate and never exceeds 2%. LM micrographs were taken with a fluorescence LM Nikon Eclipse 80i equipped with an illuminator Hg-C HGFIE of 130 W and DXM 1200F digital camera (Nikon). Abbreviations: Ctrl = control PBLs, SMF = PBLs exposed to 6-mT SMF, cycloheximide (CHX) = PBLs treated with 10-mM CHX, SMF + CHX = PBLs exposed to SMF and treated simultaneously with CHX, h = hours and bars = 10 μm.

Alterations of morphology were associated with altered cell viability. Cell viability was assayed by trypan blue dye exclusion assay and 3-(4,5-dimethylthiazol-2-yl)-2,5-diphenyltetrazolium bromide (MTT) assay, which give indication of living cells by considering plasma membrane integrity and mitochondrial activity respectively. As shown in Figure 1A, viable PBLs decreased exponentially over time. Viable SMF-exposed PBLs were always less than control ones (*p* < 0.05). The lowest values of viable cells were found at 72 h of CHX treatment. The simultaneous treatment (SMF + CHX) mitigated the deadly effects of the CHX (highest protection at 24 h), even though apoptotic and necrotic cell phenotypes were frequently found.

Representative light microscopy (LM) micrographs of PBL phenotypes (H&E staining or annexin V-FITC/propidium iodide labeling) are shown in Figure 1B–C. The count of viable, apoptotic and necrotic PBLs, done on LM micrographs of annexin V-FITC/propidium iodide labeling cells, is reported in Figure 1D. 40% of spontaneous apoptosis was measured at 72 h in control cells. Over time and in all treatment conditions, the percentage of secondary necrosis increased, and, as a consequence, apoptosis decreased.

### 2.2. Effects of 6 mT SMF

#### 2.2.1. Plasma Membrane GD3

**Figure 2:**
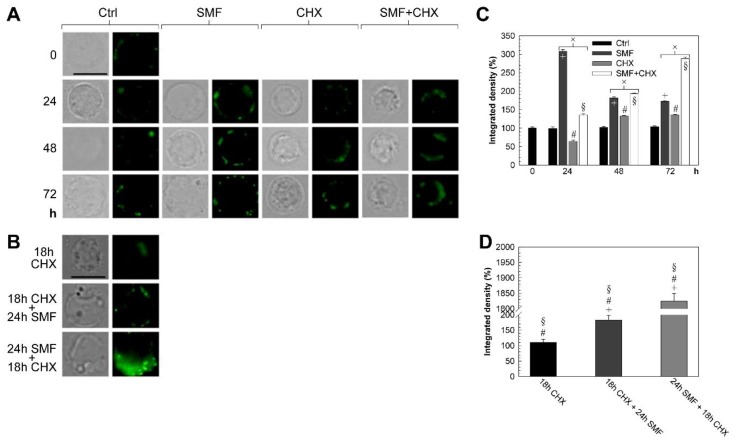

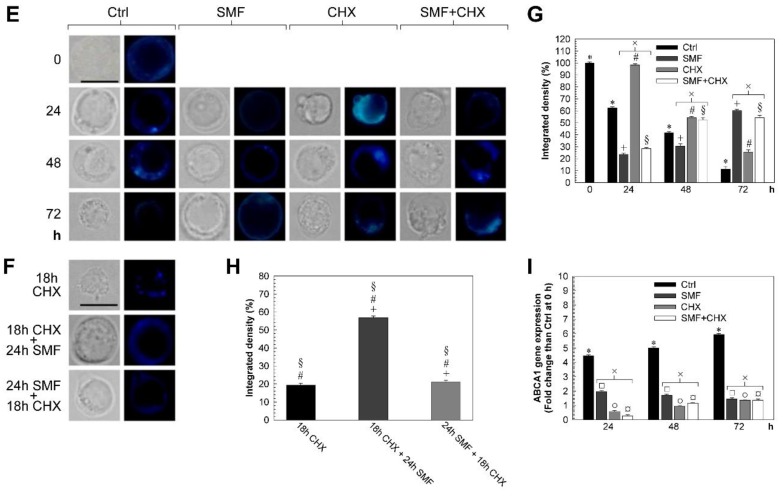
Fluorescence staining of GD3 and cholesterol and ABCA1 gene expression. (**A**–**B**, **E**–**F**): LM micrographs of PBLs following different treatments and labeled with anti-GD3 (A–B, GD3, green) or filipin (E–F, cholesterol, blue), taken with a fluorescence LM Eclipse 80i equipped with an illuminator Hg-C HGFIE of 130 W and DXM 1200F digital camera (Nikon), by setting a bright-field or a green (A–B, GD3)/blue (E–F, cholesterol) filter. (**C**–**D**, **G**–**H**): Density integrated in the green (C–D, GD3)/blue (G–I, cholesterol) channel fluorescence of LM micrographs quantified by using the image software ImageJ (US NIH) (left). In each experiment, at least 500 cells were scored. (**I**): ABCA1 gene expression levels (RT-qPCR) of PBLs following different treatments than control at the baseline time-point (0 h), by considering the 18S rRNA housekeeping gene as an internal control. The error bar represents the SE of five independent experiments, each performed in duplicate. All values plotted relate to the value of control PBLs at time 0 h (T0), taken as 100%. × indicates a significant value than control (*p* < 0.05), whereas □, ○, ¤ (*p* < 0.0167), * (*p* < 0.0083), + (*p* < 0.005) and # and § (*p* < 0.0033) indicate significant values compared to those indicated with the same symbol. Abbreviations: Ctrl = control PBLs, SMF = PBLs exposed to 6-mT SMF, CHX = PBLs treated with 10-mM CHX, SMF+CHX = PBLs exposed to SMF and treated simultaneously with CHX, 18-h CHX + 24-h SMF = PBLs exposed for 24 h to 6-mT SMF following treatment with CHX (10 mM) for 18 h, 24-h SMF + 18-h CHX = PBLs treated with CHX (10 mM) for 18 h following exposure for 24 h to 6-mT SMF, h = hours and bars = 10 μm.

LM micrographs of PBLs immunolabeled with anti-GD3 and the quantification of fluorescence as density integrated in the green channel are shown in Figure 2A–D.

GD3, always found as distributed fluorescent spots on the plasma membrane, was scarcely observed in control PBLs at T0 and it increased with time. The fluorescence signal of GD3 in SMF-exposed cells, homogenously distributed as brilliant fluorescent spots on the plasma membrane, was higher than fluorescence of control (fluorescence signal 3.1 times at 24 h and 1.7 times at 72 h *vs* control *p* < 0.05). On the contrary, in CHX-treated cells, GD3 was always found inside the blebs, while fluorescence intensity decreased at 24 h of treatment (0.6 times *vs.* control) (*p* < 0.05) and increased at 48 and 72 h with respect to control (1.3 times) (*p* < 0.05). When PBLs were simultaneously SMF-exposed and CHX-treated, GD3 was highly fluorescent with a distribution similar to CHX-treated-only cells.

Interestingly, when SMF and CHX were administered one after the other, the cell response was dependent on the sequence of administration. In fact, if PBLs were 24 h exposed to 6 mT SMF and then treated with CHX (10 mM) for 18 h, the fluorescence intensity and distribution were very similar to CHX alone treatment. On the contrary, when CHX (10 mM) was given for 18 h before 24 h exposure to 6 mT SMF, GD3 fluorescence intensity and distribution was similar to the SMF alone treatment.

#### 2.2.2. Cytoplasmic and Plasma Membrane Cholesterol

LM micrographs of PBLs immunolabeled with filipin and the quantification of fluorescence as density-integrated in the blue channel are shown in Figure 2E–H. In control cells, cholesterol was observed inside the cells and mostly on the plasma membrane after 48 h of culture. The fluorescence signal decreased with time and, at 72 h, was only 11.43% *vs* control at T0 (*p* < 0.0083). In the SMF-exposed PBLs, the cytoplasmic and plasma membrane cholesterol were lower than control up to 48 h (lowest value at 24 h) and the fluorescence intensity increased above the control at 72 h. When cells were treated with CHX alone or simultaneously with SMF, cholesterol localized mainly in the blebs of the plasma membrane. By increasing time of treatment, the cholesterol decreased in cells treated with CHX but increased when PBLs underwent the double treatment.

When SMF and CHX were administered one after the other, the cell response was dependent on the sequence of administration. In fact, when PBLs were first exposed for 24 h to 6-mT SMF and then treated with CHX (10 mM) for 18 h, the fluorescence intensity and distribution of cholesterol was similar to CHX alone treatment. Conversely, when CHX (10 mM) for 18 h was given before 24-h exposure to 6-mT SMF, the expression and distribution of cholesterol was similar to the SMF alone treatment. Fluorescence was generally observed distributed all over the plasma membrane and inside blebs, while in the presence of CHX (alone or in combination with SMF), the fluorescence was observed mainly in the blebs free plasma membrane.

#### 2.2.3. ABCA1 Gene Expression

ABCA1 gene expression levels (RT-qPCR) of PBLs at the different treatments are shown in Figure 2I. In control cells, a time dependent up-regulation of ABCA1 gene expression (six times higher than T0 after 72 h of culture) was measured, in the treated cells, ABCA1 gene expression was down-regulated when compared to control. A progressive decrement of ABCA1 gene expression with time of treatment was observed in SMF exposed cells, while, after a dramatic down- regulation at 24 h of CHX treatment ABCA1 gene expression slowly increased, but values were always lower than the control.

#### 2.2.4. Colocalization of GD3/Cholesterol in Plasma Membrane Lipid Rafts and Nuclear Alterations

**Figure 3:**
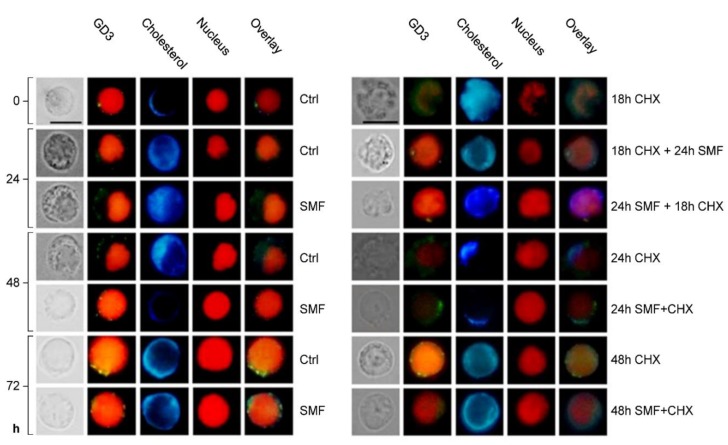
Triple-fluorescence staining of GD3, cholesterol and nucleus. LM micrographs of PBLs following different treatments and labeled with anti-GD3 (GD3, green); filipin (cholesterol, blue) or propidium iodide (DNA, red) taken with a fluorescence LM Eclipse 80i equipped with an illuminator Hg-C HGFIE of 130 W and DXM 1200F digital camera (Nikon), by setting a bright-field or a green (GD3)/blue (cholesterol)/red (double-stranded DNA) filter. Abbreviations: Ctrl = control PBLs, SMF = PBLs exposed to 6-mT SMF, CHX = PBLs treated with 10-mM CHX, SMF+CHX = PBLs exposed to SMF and treated simultaneously with CHX, 18-h CHX + 24-h SMF = PBLs exposed for 24 h to 6-mT SMF following treatment with CHX (10 mM) for 18 h, 24-h SMF + 18-h CHX = PBLs treated with CHX (10 mM) for 18 h following exposure for 24 h to 6-mT SMF, h = hours and bars = 10 μm.

The distribution of the lipid rafts on the plasma membrane and the nucleus morphology of PBLs during the different treatments were monitored by the simultaneous labeling of Ab anti-GD3, filipin and propidium iodide (Figure 3). Discrete lipid rafts were found in control PBLs at the longest time of culture. SMF exposure (also simultaneously with CHX treatment) favors GD3 and cholesterol to co-localize in discrete microdomains whose fluorescence was very intense at 72 h. Interestingly, lipid rafts preferentially localized in correspondence to greater presence of cytoplasm. An opposite scenario was observed for CHX- treated PBLs, in which fluorescence was scarce and of low intensity.

Condensed chromatin was largely observed in 18 h CHX-treated PBLs nuclei, but was reduced when 18 h CHX treatment was followed by 24 h SMF exposure. Viceversa, i.e., SMF given before CHX treatment, gave rice to many nuclear changes, that were not observed in control or SMF exposed cells.

### 2.3. ROS Production and Plasma Membrane Lipid Peroxidation

**Figure 4:**
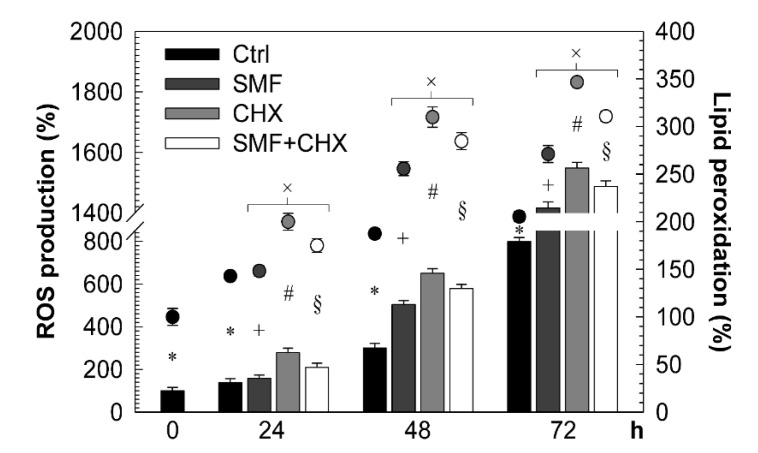
ROS production and lipid peroxidation. ROS production and lipid peroxidation of PBLs following different treatments, quantified by performing the nitro blue tetrazolium (NBT) assay (histograms) and ThioBarbituric Acid (TBA) assay (dots). All values plotted relate to the value of control PBLs at the time-point 0 h, taken as 100%. The error bar represents the SE of five independent experiments, each performed in duplicate. × indicates a significant value than control (*p* < 0.05), whereas * (*p* < 0.0083) and +, # and § (*p* < 0.0167) indicate significant values compared to those indicated with the same symbol. Abbreviations: Ctrl = control PBLs, SMF = PBLs exposed to 6-mT SMF, CHX = PBLs treated with 10-mM CHX, SMF + CHX = PBLs exposed to SMF and treated simultaneously with CHX and h = hours.

Regardless of the treatments, ROS production and membrane lipid peroxidation increased with time (Figure 4).

The type of treatment determined the amount of increment: the highest values were measured for CHX-treated cells and the lowest for controls (CHX > CHX + SMF > SMF > Ctrl).

### 2.4. Uptake of AgNPs-G

Freshly synthesized AgNPs-G are relatively monodispersed in size (the size distribution ranges from 20 to 40 nm, and the average size is d = 30 nm, with a standard deviation of 5 nm) with spherical shapes. The capping of glucose is stable up to 10 days, since during the immersion tests, the glucose as well as total Ag^+^ in the culture medium do not increase (data not shown). After 10 days in Roswell Park Memorial Institute (RPMI) 1640 culture medium, AgNPs solution loses stability and an increasing number of nanorods (average longitudinal diameter = 50 nm; standard deviation = 25 nm) are present.

ICP-OES (Inductively Coupled Plasmon Optical Emission Spectroscopy) allows to evaluate for each time-point the silver ions in the culture medium and in cell extracts, giving an indirect quantification of internalized AgNPs. On the basis of our previous study, we use 2 × 10^3^ β-d glucose-coated AgNPs/cell that do not affect viability of the PBLs [9].

**Figure 5:**
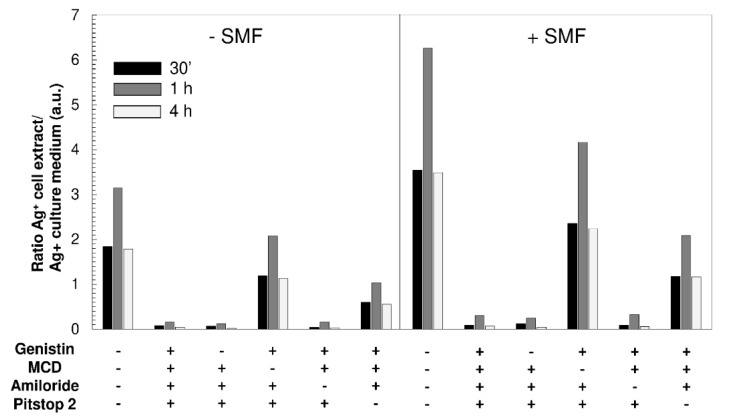
Inductively coupled plasmon optical emission spectroscopy (ICP-OES) analysis of the amount of silver nanoparticles synthesized using β-d-glucose (AgNPs-G) internalized by PBL cells previously exposed to SMF for 72 h and treated with endocytic pathway selective inhibitors (genistin, MCD, amiloride and Pitstop 2) as reported in Section 4.7 of Materials and Methods were further incubated with 2 × 10^3^AgNPs-G for 30 min, 1 h and 4 h. Data are reported as a ratio between the Ag^+^ detected in cell extracts and those detected in the culture medium (absorbance in arbitrary unit, a.u.) from 3 independent experiments. Abbreviation: SMF = PBLs exposed or not exposed to 6-mT SMF. (+) presence and (−) absence.

Aiming to investigate the mechanisms involved in Ag-NPs uptake, some selective inhibitors of different endocytic pathways, as reported in the Materials and Methods section, were used to evaluate the AgNPs uptake efficiency and the possible effects of 6-mT SMF exposure. Data reported in Figure 5 suggest that AgNPs-G uptake by PBLs involves lipid raft- and clathrin-mediated endocytosis. Seventy-two hours of 6 mT SMF exposure before AgNPs-G incubation favors their internalization. The efficiency of uptake is high at 1 h of AgNPs-G incubation. The pre-treatment with MCD and pitstop 2, which inhibit lipid raft-mediated and clathrin-mediated endocytosis, respectively, significantly decreased the amount of internalized AgNPs-G. Moreover, the internalization of AgNPs-G via lipid rafts is more efficient (of about 60%) than via clathrin pits. Genistin and amiloride were ineffective to modulate AgNPs-G uptake, thus indicating that caveolae and micropinocytosis pathways are not involved. Regardless of incubation time and inhibitors used, the exposure to 6 mT SMF for 72 h before the incubation with AgNPs-G increases of about 50% the efficiency of internalization (Figure 5). Similar results were achieved by exposing PBLs to 24 h of 6 mT SMF before incubation with AgNPs (Figure S3).

## 3. Discussion

It was here shown that 6 mT SMF, when applied in vitro to PBLs, induced a cell redistribution of GD3 and cholesterol that, in turn, positively impacts the AgNPs-G internalization. In particular, SMF induces GD3 and cholesterol to concentrate in microdomains of the plasma membrane, the lipid rafts, in a time dependent way, by moving concomitantly the cholesterol from cytoplasm into the plasma membrane. Lipid rafts of the plasma membrane were particularly abundant in the 72 h SMF exposed PBLs. The evaluation of internalization mechanisms suggested that AgNPs-G preferentially utilized raft domains and clathrin pits to pass PBLs plasma membranes. Moreover, the rearrangement of lipid raft localizations and abundances on the plasma membrane, following exposure of the PBLs to 6-mT SMF for 72 h before adding AgNPs-G, induced an increase of the efficiency of internalization.

Since one of the general functions of lipid rafts is to separate proteins along the plasma membrane [19], the lipid raft localization after 72 h of SMF exposure at the plasma membrane in correspondence to the greatest cytoplasm content may be a mechanism activated by SMF to compartmentalize specific proteins involved in the signal transduction, by moving within the lipid rafts only proteins to be activated. The great amount of plasma membrane lipid rafts observed in SMF exposed PBLs could be a mechanism activated by SMF to recruit specific plasma membrane proteins. We and other groups have already demonstrated the influence of SMF on plasma membrane proteins. In fact, 24 h of 6 mT SMF exposure affects the plasma membrane calcium channels through the Ca^2+^/H^+^ antiporter, which, in turn, leads to the extrusion of H^+^ ions outside of the cell. The same H^+^ extrusion observed under SMF exposure could occur following the induction of the Na^+^/H^+^ antiporter mainly involved in the regulation of the intracellular pH of lymphocytes [11]. SMF down-regulated the human membrane protein ABCA1, regulating cholesterol efflux, which decreased with increasing exposure times. Thus, the highest amount of cholesterol observed in both the cytoplasm and plasma membrane of 72-h SMF-exposed PBLs could be due to the lower levels of expression of the ABCA1 gene, leading to less cholesterol efflux. Increased levels of cholesterol found in response to longer SMF exposures could be an adaptive response for cellular protection [20].

SMF exposure did not induce significant cell death or lipid peroxidation. These data are in line with reports showing the absence of cell death or end-products of lipid peroxidation of the plasma membrane in 7 mT SMF exposed mouse PBLs for 3 h. The fact that lower GD3 expression is observed with increasing SMF exposure times appears to be related to increased cytoplasmic ROS production, which negatively affects the expression of the GD3 synthase gene [21].

SMF interferes with the ROS chemistry by changing the spin of electrons and induces conformational changes of antioxidant enzymes, which therefore lose their catalytic activity [22]. Interestingly, GD3 (decrease) and ROS (increase) were seen vary by increasing the time of exposure to SMF. This finding is partially in accord to the correlation between the expression of the GD3 synthase gene and the generation of ROS [21].

The effects of SMF on the biochemistry of cells are correlated with concomitant morphological changes. In fact, the percentage of lymphocytes bearing shape modifications increased with the time of SMF exposure, mainly losing the rounded shape. The change of spins of electrons of free radicals under SMF, by interfering with their coupling mechanisms, led to changes in the kinetics of chemical reactions that, in turn, altered the cell physiology and, as a result, of the cytoskeletal rearrangements and/or through direct influence on structural components of the plasma membrane [23,24]. In addition, certain cytoskeleton components are closely associated with lipid rafts [25], and thus, rearrangements of lipid rafts affect cell shape by cytoskeleton modifications. In addition, SMF induced the cell redistribution and colocalization of GD3 and cholesterol in plasma membrane lipid rafts in an exposure time dependent way. Interestingly, the GD3 and cholesterol always localized on the plasma membrane of PBLs, enclosing the largest amount of cytoplasm.

Finally, our data suggest that the expression/distribution of the disialoganglioside GD3 and cholesterol on the plasma membrane of PBLs were affected not only by SMF exposure but also by apoptotic induction. This is in agreement with literature data about T cells, in which the activation of cell death signals depends strongly on the association of the FAS receptor and lipid rafts at the plasma membrane [26,27]. GD3 plays a role in apoptotic machinery, acting as an intracellular pro-apoptotic lipid messenger moving into vesicles that detach from the plasma membrane toward the mitochondria. Then, these vesicles induce a gradual depolarization of the membrane potential (Δψ_m_), which, in turn, trigger apoptosis through the release of AIF and cytochrome c [28]. Thus, it could be possible that SMF fulfills its anti-apoptotic action in PBLs during exposure, causing GD3 to move from plasma membrane to cytoplasm.

Based on the results reported above, our idea is to evaluate the possibility to rearrange lipid rafts by using SMF exposure to modulate AgNPs uptake by lymphocytes. In fact, there are reports on the application of metallic NPs for diagnostic purposes, such as leukocytes immunophenotyping [29,30], so, understanding the uptake mechanisms of NPs and the modality to favor the internalization could be pivotal in medicine. It has been widely demonstrated that the uptake of AgNPs depends on both the physicochemical features of nanoparticles and cell type, and the mechanism of internalization is a key factor in terms of uptake efficiency [4]. Previous data achieved by us suggest that a low AgNPs-G amount (2 × 10^3^ NPs/cell) had no toxic influence on lymphocytes, since β-d-glucose-capping ensures very low dissolution of Ag^+^ from AgNPs, and no loss of glucose was observed in the RPMI 1640 culture medium; moreover, we have observed a culture time- and concentration-dependent absorption/uptake of AgNPs [9]. AgNPs-G could be a novel nanoscale system to deliver drugs to lymphocytes, in addition to other NPs [31], and understanding the mechanism of NP uptake by cells is very important for drug and gene delivery [32]. Literature data suggest that NPs can enter cells by choosing among different endocytosis mechanisms [4] in relation to their size and cell type. Wiwanitki and coworkers reported that AuNPs enter into lymphocytes by direct penetration into the cytoplasm, since they [33] have no phagocytosis activity. However, the reported pore sizes in lymphocyte membranes (of about 4 × 2.5 nm) is smaller than the diameter of NPs used in this study (30 ± 5 nm), as well as in diagnostics. Here, we demonstrated that uptake of AgNPs-G by PBLs involves lipid raft- and clathrin-mediated endocytosis, and the internalization via lipid rafts is more efficient (of about 60%) than via clathrin pits. Further, the exposure to 6-mT SMF for 72 h before the incubation with AgNPs increases of about 50% the efficiency of internalization.

From all the above reported, it derives that SMF induces, in an exposure time-dependent way, the cell redistribution of GD3 and cholesterol, which co-localize in plasma membrane lipid rafts; accordingly, the expression of the ABCA1 gene, encoding for a membrane-associated protein that mediates the efflux of cholesterol, decreases. Moreover, we provide the demonstration that AgNPs-G enter lymphocytes through lipid raft- and clathrin-mediated endocytosis, and the preliminary exposure to SMF favors the internalization.

## 4. Materials and Methods

### 4.1. Ethics Statements

Human blood samples of healthy donors were obtained by buffy coats supplied by the Hospital ‘S. Giuseppe da Copertino’, Lecce, Italy, according to national guidelines of the Italian National Committee of Bioethics. The identity of the donors remained anonymous. The need of donor consent was waived by the ethics committee. The use of buffy coat was acknowledged by the “Comitato Etico dell’ASL LE” (Ethics Committee of the Health Service of Lecce), Lecce, Italy. This ethics committee is an independent organization that is working under the Declaration of Helsinki and following the rules of Good Clinical Practices, according to international and national laws and to the guidelines of the Italian National Committee of Bioethics.

### 4.2. Chemicals

All chemicals were of analytical grade and, unless otherwise indicated, were purchased from Sigma-Aldrich (Sigma, St. Louis, MO, USA).

### 4.3. Cell Cultures

Peripheral Blood Mononuclear Cells (PBMCs, monocytes and lymphocytes) were isolated by Ficoll gradient separation from human buffy coats of both nonsmoker male and female donors, healthy and aged between 25 and 45. A double-gradient centrifugation in Ficoll and a double-adherence to plastic were used to obtain a 95% pure cell culture. The density gradient centrifugation using Ficoll yields a white interphase containing the peripheral blood mononuclear cells (PBMCs, monocytes and lymphocytes). This was confirmed by a May-Gruenwald staining of the collected cells, which show both a high nucleus/cytoplasm ratio (typical of lymphocytes) and bean-shaped nuclei (typical of monocytes).

Monocytes were depleted from the isolated mononuclear cell suspension by taking advantage of the fact that monocytes adhere to plastic, whereas lymphocytes do not. So, the cell suspension of the PBMCs was incubated horizontally for 1 h in a 37 °C, 5% CO_2_ humidified incubator. After this, nonadherent lymphocytes were centrifuged 10 min at 450 to 600× *g*, 18 °C to 20 °C. The supernatant was removed, and the cells were resuspended in complete roswell park memorial institute (RPMI) 1640 medium. The viability of isolated lymphocytes was evaluated by the trypan blue dye exclusion test.

PBLs were cultured in 25 cm^2^ flasks or 8 cm^2^ Petri dishes (Iwaki, Tokyo, Japan), at a cell density of 10^6^ cells/mL in RPMI 1640 medium (Cambrex Bio Science, Verviers, Belgium) supplemented with 10% (*v*/*v*) inactivated fetal calf serum (Cambrex Bio Science, Verviers, Belgium), 2 mM L-glutamine (Cambrex Bio Science, Verviers, Belgium), 100 IU/mL penicillin and streptomycin in a humidified atmosphere of 5%-CO_2_ at 37 °C. PBLs were used for all investigations 24 h after their isolation, which was considered as the baseline time point 0 (0 h). PBLs were continuously exposed for up to 72 h to a uniform 6-mT SMF and/or treated with 10-mM cycloheximide (CHX). Control cells were cultured in the same culture condition without receiving SMF exposure and apoptotic inducer. Control samples were exposed only to the geomagnetic field, magnetic induction whose values were about 3 orders of magnitude lower than the exposed ones (see Section 4.4). The biochemical and morphological investigations were done at fixed times, from 18 h to 72 h of culture for the different treatments.

### 4.4. SMF Exposure

Cells were exposed to SMFs by using the “Magnetostatic Field System for Exposure of Cell Cultures”, Ma.Fi.S.E.C., better descripted elsewhere [34]. Briefly, two NdFeB magnetic rectangular plates, sized 135 × 100 × 2 mm, supplied by China Rare Earth Magnetic Co Ltd. (Nanshan District, Shenzhen, China) and coated with Ni, grade N35, *B_r_* 1170-1220 mT, magnetized through the thickness and separated by air as a dielectric medium, were fixed in an inert materials’ support structure (plexiglass, nylon-66 and polyvinyl chloride). These materials do not disturb the magnetic field configuration and are inert to ultraviolet (UV) rays, which were used to sterilize the Ma.Fi.S.E.C. The culture flask/Petri dish was positioned on the middle shelf and was between the upper and bottom magnetic rectangular plates, mounted with opposite polarity at a distance of 80 mm from the middle shelf. This organization allowed a uniform 6-mT magnetic induction inside the culture flask/Petri dish. PBLs cultures were always placed on the same two shelves of the cell culture incubator, where the ambient 50-Hz magnetic field was 0.95/0.62 µT (heater on/off). In the experimental room where the physical measures were taken, the background magnetic induction was 10 µT (static), while in the environments where the cells were processed (i.e., laboratory, incubators, worktops and cell culture hood), the magnetic field measures ranged between 0.08 and 0.14 µT (50 Hz). The local geomagnetic field was approximately 43 µT; no other significant effect, by including any temperature rise, was detected throughout 72 h.

Magnetic field intensity was measured by using a digital gaussmeter (Model CI-6520A; PASCO Scientific, Roseville, CA, USA) with a sensitivity range of ±10 mT, 5 μT of resolution and 1 mT of accuracy, connected to a graphic interface (Science Workshop 750 interface; PASCO Scientific). The errors for all measures never exceeded 2%.

### 4.5. Biochemical and Molecular Biology Investigations

#### 4.5.1. Viability assay: 3-(4,5-dimethylthiazol-2-yl)-2,5-diphenyltetrazolium Bromide (MTT) Assay and Trypan Blue Dye Exclusion Test

MTT is reduced to formazan crystals only by mitochondria of living cells. Thus, the quantification of these crystals is considered as an indirect measurement of cell viability [35]. Briefly, 5 × 10^5^ PBLs were incubated with 1 mg/mL of MTT (*w*/*v* in supplemented RPMI 1640) for 2 h at 37 °C and 5% CO_2_. First the cells were rinsed three times with phosphate buffer saline (PBS) (0.2 M), pH 7.4, then the formazan crystals were solubilized with dimethylsulfoxide (DMSO; Carlo Erba, Milan, Italy), and the absorbance of obtained colored solution was read by using an Ultrospec 4000 UV/visible spectrophotometer (Pharmacia Biotech, Stockholm, Sweden) set at 570 nm.

Viability was expressed as a percentage of the relative growth rate (RGR) by Equation 1:

RGR = (D_sample_/D_control_) × 100 (1)

where *D_sample_* and *D_control_* are, respectively, the absorbance (at 570 nm) of the test samples and the negative controls.

The trypan blue dye exclusion test was performed by mixing 10 µL of PBL suspension with 90 µL of filtered 0.5% trypan blue (*w*/*v* in Krebs solution). The percentage of viable cells was scored by using a Bürker chamber (Blaubrand, Germany) and was calculated as follows (Equation 2):

Viable cells (%) = (Average number of viable cells per mL of aliquot/Average number of cells per mL of aliquot) ×100(2)

#### 4.5.2. Nitro Blue Tetrazolium (NBT) Assay

The reduction of the NBT salt to diformazan crystals is considered as an indirect measurement of the intracellular ROS production [36]. PBLs (5 × 10^5^) were incubated with 335-μg/mL NBT (*w*/*v* in supplemented RPMI 1640) for 2 h at 37 °C and 5% CO_2_, then rinsed three times with absolute methanol (Carlo Erba). The absorbances of diformazan crystals, firstly dissolved with a freshly prepared 2-M KOH/DMSO solution, were read by using an Ultrospec 4000 UV/visible spectrophotometer (Pharmacia Biotech, Stockholm, Sweden) set at 630 nm.

#### 4.5.3. Thiobarbituric Acid (TBA) Assay

Polyunsaturated fatty acids generate malondialdehyde (MDA) upon oxidative decomposition. Thus, the adduct 1:2 that one molecule of MDA forms with two molecules of TBA is considered as an indirect measurement of plasma membrane lipid peroxidation [37]. PBLs (15 × 10^6^) were rinsed three times with filtered PBS (0.2 M), pH 7.4, and sonicated for four cycles on ice by setting for each cycle 40% of amplitude, 10 s of sonication and 5 s of pause (Sonoplus Ultrasonic homogenizer HD 2070; Bandelin Electronic, Berlin, Germany). Proteins were precipitated by adding cold 10% (*w*/*v* in water) trichloroacetic acid, then cell lysate was incubated with 16-mM TBA (prepared in 10-μM NaOH) in a water bath at 90 °C for 45 min. Reaction was stopped by placing the reaction mix on ice for 5 min. After another 5 min at room temperature (RT), the 1:2 adduct was extracted by using 500 μL of *n*-butanol and 50 μL of a saturated solution of NaCl (JB Baker, Deventer, Holland). Samples were centrifuged at 12 × 10^3^ rpm for 1 min., then the absorbance of the supernatant was read by using an Ultrospec 4000 UV/visible spectrophotometer (Pharmacia Biotech, Stockholm, Sweden) set at 532 nm. A positive control of reaction 0.5-mM MDA was used instead of cell lysate.

#### 4.5.4. ABCA1 Gene Expression by Real-Time Quantitative Reverse Transcription Polymerase Chain Reaction (qRT-PCR)

Total RNA extraction was performed using the TRIZOL reagent, according to the manufacturer’s instructions (Invitrogen, Carlsbad, CA, USA). The quality and quantity of the purified RNA were analyzed using a NanoSpectrophotometer (Epoch, BioTek, Bad Friedrichshall, Germany); then, the integrity of each RNA sample was examined by using 1% agarose gel, 1× TBE. RNA was converted to cDNA using a ThermoScript RT-PCR System (Invitrogen Life Technologies, Carlsbad, CA, USA).

According to the 2-ΔΔCt method, the threshold cycle (Ct) values generated by the CFX Manager™ software (BioRad, Hercules, CA, USA) were used to analyze the ABCA1 gene expression levels of PBLs following different treatments than control at the baseline time-point (0 h), by considering the 18S rRNA housekeeping gene as an internal control. qRT-PCR amplification was carried out on the BioRad CFX96 Real-Time PCR System C1000 Thermal Cycler by using the SYBR^®^ Premix Ex Taq II Kit (TakaRa Bio Inc., Kusatsu, Shiga, Japan—No. RR820L), according to the manufacturer’s instructions, including forward primer (5’-TGCAAGGCTACCAGTTACATT-3’) and reverse primer (5’-TTAGTGTTCTCAGGATTGGCT-3’). Following an initial 30-s denaturation at 95 °C, 40 cycle amplifications were performed with denaturation at 95 °C for 5 s, annealing at 60 °C for 30 s, extension at 72 °C for 30 s and a final elongation by performing a temperature ramping from 72 °C to 95 °C for 0.5 °C/0.05 s.

### 4.6. Immuno- and Cytochemistry

#### 4.6.1. Labeling of Apoptotic and Necrotic Cells

The evaluation of viable, apoptotic and necrotic cells was performed following the protocol indicated by the producer of the Annexin V-FITC Apoptosis Detection Kit (Sigma-Aldrich., Cat. No. APOAF, St. Louis, MO, USA). Briefly, cells following an extensive washing with PBS were incubated with a solution containing annexin V-FITC and propidium iodide (500 μL of binding buffer 1× + 5 μL of annexin V-FITC + 10 μL of propidium iodide). Cells were rinsed twice with 0.2M of PBS (pH 7.4) and incubated for 10 min in the complete culture medium containing 0.5 mg/mL of FITC-conjugated annexin-V and 2 mg/mL of PI. Early apoptotic and apoptotic cells were recognized as annexin-V-positive using a fluorescence microscope Eclipse 80i (Nikon, Kawasaki, Kanagawa Prefecture, Japan). Necrotic cells were simultaneously stained by PI and annexin-V–FITC, while viable cells were not stained.

#### 4.6.2. Single or Co-Labeling of Disialoganglioside GD3, Cholesterol and Double-Stranded DNA

PBLs (15 × 10^6^) rinsed three times with PBS (0.2 M), pH 7.4, were incubated for 30 min with 10 μg/mL of monoclonal mouse IgM anti-GD3 (Seikagaku Corporation, Chiyoda-ku, Tokyo, Japan) and for an additional 30 min with 22 μg/mL of anti-mouse IgG-FITC at 4 °C. To avoid the unspecific antigen recognition, the Ig solutions were prepared in PBS (0.2 M), pH 7.4, added with 1% bovine serum albumin (*w*/*v*). Cells were fixed for 1 h with freshly prepared 3% (*v*/*v* in PBS (0.2 M), pH 7.4) formaldehyde (Carlo Erba) that was then quenched for 10 min with 1.5 mg of glycine/mL of PBS (0.2 M), pH 7.4, at RT. The cholesterol was labeled for 2 h with a 0.05-mg filipin (Cayman Chemical, Milan, Italy)/mL of PBS (0.2 M), pH 7.4, added with 10% of FBS. DNA was labeled for 10 min with 2% propidium iodide (*v*/*v*, in binding buffer 1×) at RT. Finally, cells were extensively washed before their observation.

Light microscopy (LM) micrographs were taken with an epifluorescence light microscope Nikon 80i equipped with a C-HGFIE Hg-precentered fiber illuminator (130 W Hg lamp) and a digital camera DXM 1200 F (Nikon, Kawasaki, Kanagawa Prefecture, Japan), by setting the suitable filter to detect the specific emission wavelength (λ_em._ in nm) of the fluorophore probe (λ_em.filipin_ = 480, blue filter; λ_em,FITC_ = 525, green filter and λ_em.propidium iodide_ = 617, red filter). In order to minimize photobleaching, all labeling was performed in a dark room, and all images were taken within 5 min following labeling. Fluorescence, as density-integrated in the green (FITC)/blue (filipin) channel, was quantified by using the analysis image software ImageJ (US NIH, Bethesda, MD, USA).

### 4.7. Uptake of AgNPs-G

AgNPs-G were obtained and characterized as reported in Panzarini et al., 2017. Briefly, AgNPs-G were obtained by adding 2 mL of a 10^−2^ M aqueous solution of AgNO_3_ to 100 mL of a 0.3-M β-d-glucose water solution. The mixture was boiled for 30 min under vigorous stirring. Yellow color of the solution indicated the formation of AgNPs. The average and distribution size, morphology and stability of the NPs have been studied by high-resolution transmission electron microscopy (TEM) and UV–visible spectroscopic techniques. Transmission electron microscopy (TEM) observations were performed by a Hitachi 7700 at 100 kV (Hitachi High Technologies America Inc., Dallas, TX, USA). Particles size distribution has been obtained using the ImageJ program (US NIH, Bethesda, MD, USA). Ultraviolet–visible (UV–Vis) spectra were recorded in the range between 300 and 800 nm by using a T80 spectrophotometer (PG Instruments Ltd., Leicester, UK). The stability of different AgNPs-G concentrations were assayed in a RPMI 1640 culture medium up to 10 days. The dissolution of AgNPs-G, in terms of the release of Ag^+^ up to 10 days in a RPMI 1640 culture medium, was determined by atomic absorption spectroscopy (AAS; Thermo Electron Corporation, M-Series, Thermo Fisher Scientific, Waltham, MA, USA) after precipitation of AgNPs-G by ultracentrifugation (24,900 g; 30 min at 4 °C). The detection limit was 1 μg/L. The stability of the capping of glucose was evaluated by sugar quantification up to 10 days in a RPMI 1640 complete culture medium via a Spectrophotometric Glucose Assay kit, ab65333 (Abcam, Cambridge, UK).

PBLs were incubated with 2 × 10^3^AgNPs-G/cell after the exposure of cells to 6-mT SMF for 72h. Before the incubation with AgNPs, the PBLs were centrifuged to remove the apoptotic cells and debris.

For the endocytosis inhibitors, the PBLs were incubated with 200 μM of genistein (caveolae), 2 mM of MCD (methyl-β-cyclodextrin) (lipid rafts), 1.5 mM of amiloride (micropinocytosis) and 12.5 μM of pitstop 2 (clathrin-coated pits). Genistein, MCD and amiloride were applied for 40 min, whereas pitstop 2 was applied for 10 min. After incubation with endocytosis inhibitors, the cells were washed and further incubated with AgNPs-G for 30′, 1 h and 4 h.

The ability of PBLs to uptake AgNPs-G was investigated by using an inductively coupled plasmon optical emission spectroscopy (ICP-OES) analysis. ICP-OES is a technique commonly used for the analysis of metals in various fields based on atomic emission spectroscopy, where the sample at high-temperature plasmas up to 8000 K is converted to free, excited or ionized ions. The ions emit a radiation when back to the ground state, whose intensities are optically measured and indicate the amount of ions.

After incubation of PBLs with AgNPs-G, the culture medium was centrifuged (5 min, 1000 rpm, 4 °C) to collect PBLs. The supernatant was frozen (24 h at −20 °C), lyophilized and stored at −80 °C until analysis. The PBLs were washed twice with PBS to remove potential medium residue and then extracted in 2 mL of perchloric acid (PCA; 0.9 mol/L). The samples were sonicated (Brooklyn Instruments, New York, NY, USA) for 60 s and then centrifuged for 15 min (1000 rpm at 4 °C). The supernatant pH values were then adjusted to 7.0 ± 0.1 using 1mol/L of KOH and 1mol/L of HCl, followed by centrifugation (3000 *g* at 4 °C) for 10 min. The resulting clear supernatants were lyophilized and stored at −80 °C until analysis. Before the ICP-OES analysis (Perkin Elmer Optima 7300 V HF version, Perkin Elmer, Shelton, CT, USA), the samples were neutralized in concentrated HNO_3_ for 5 min on a heating block. The measurements were performed against a silver standard of 1 mg/L. The results are reported as a ratio of Ag^+^ in cell extracts/Ag^+^ in the cell culture.

### 4.8. Statistical Analysis

Data were analyzed by performing a one-way analysis of variance (ANOVA) at the 95% confidence level. A post hoc Bonferroni test was performed by setting the experiment-wise error rate at 0.05 and the adjustment factor as 3, 5, 6 or 15, depending on comparations (combinations, without repetition, of n elements in groups of k, n!/[k!×(n − k)!]). Thus, differences were significant at Bonferroni-adjusted critical *p*-values (indicated for simplicity with *p*) of 0.0167 (0.05/3), 0.01 (0.05/5), 0.0083 (0.05/6), 0.005 (0.05/10) or 0.0033 (0.05/15). The error bar represents the mean ± the standard error (SE) of five independent experiments, each done in duplicate.

## 5. Conclusions

In consideration of the fact that SMFs play a pivotal role in the rearrangement of lipid rafts and their principal components, GD3 and cholesterol, they could be a useful tool to be exploited for therapeutic and diagnostic purposes in nanomedicine. The capability of AgNPs to pass the plasma membranes of PBLs confirm their exploitable use as vehicles, suitable to developing new therapeutic strategies for autoimmune diseases and new noninvasive tracking of lymphocytes. Further, the ability of 6-mT SMF to increase the uptake of AgNPs into lymphocytes opens new horizons in medicine.

## Supplementary Materials

The following are available online. Figure S1: Cell viability evaluated by MTT assay from 30 min to 72 h of PBLs supplemented with PHA (10 μg/mL). All values are referred to the value of the control PBLs at 0 h (before the start of the time course), taken as 100%. Each error bar represents the SE of five independent experiments, performed in duplicate. Figure S2: Cell viability evaluated by MTT assay from 30 min to 72 h of PBLs supplemented with PHA (10 μg/mL) in the presence and in the absence of 6-mT SMF. All values refer to the value of the control PBLs at 0 h (before the start of the time course), taken as 100%. Each error bar represents the SE of five independent experiments, performed in duplicate. Figure S3: ICP-OES analysis of the amount of AgNPs-Gs internalized by PBL cells previously exposed to SMF for 24 h, treated with endocytic pathways selective inhibitors (Genistin, MCD, Amiloride and Pitstop 2) and further incubated with 2 × 10^3^ AgNPs-G for 30 min, 1 h and 4 h. Data are reported as a ratio between the Ag^+^ detected in the cell extract and those detected in the culture medium (absorbance in arbitrary unit, a.u.). (+) presence and (−) absence.
