# Supplementary material for: Moderate Static Magnetic Field (6 mT)-Induced Lipid Rafts Rearrangement Increases Silver NPs Uptake in Human Lymphocytes
Source: Molecules. 2020 Mar 19;25(6):1398. doi: 10.3390/molecules25061398 (PMC7144570; doi:10.3390/molecules25061398)
Supplement: Supplementary file 1 [file molecules-25-01398-s001.pdf]

molecules

MDPI

Supplementary Material

Cultures of isolated PBLs show a progressive decrement due to spontaneous cell death unless they are supplemented with a mitogen, like PHA, able to transform large population of lymphocytes in mitotically active cells, and thus, they can be maintained in culture up to 1 week by preventing cell death. Without PHA about 20% of untreated PBLs died. However, if the trend of PBL viability is measured in the presence or absence of PHA (10µg/mL), the viability values are almost similar up to 18 h. The PHA supplemented PBLs remain more viable for the remaining days (Figure 1 Supplementary material).

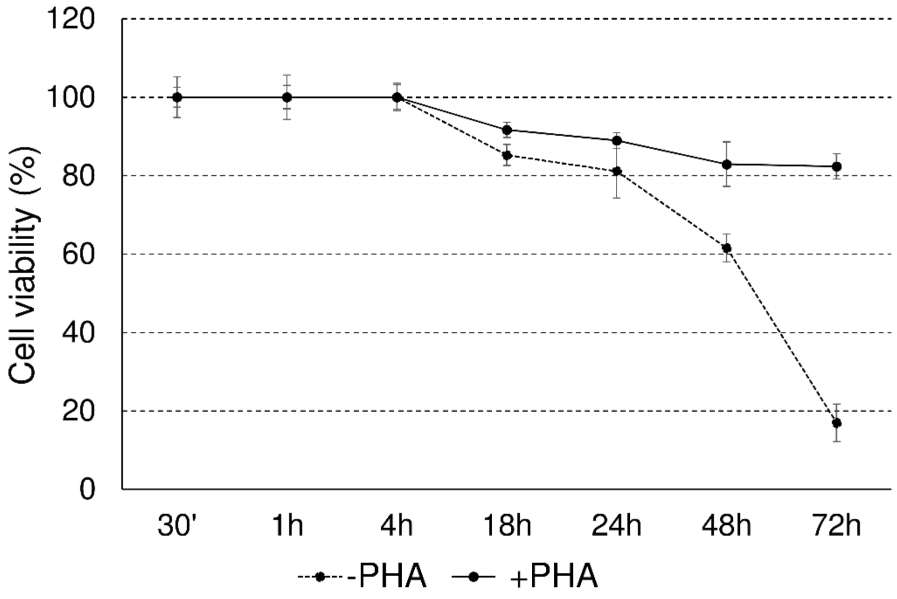

Figure 1 Supplementary material. Cell viability evaluated by MTT assay from 30 minutes to 72 h of PBLs supplemented with PHA (10µg/mL). All values are referred to the value of control PBLs at 0 h (before the start of time course), taken as 100%. Each error bar represents the SE of five independent experiments, performed in duplicate.

The exposure to 6 mT SMF of PHA treated PBLs does not improve cell viability of PBLs (Figure 2 Supplementary material).

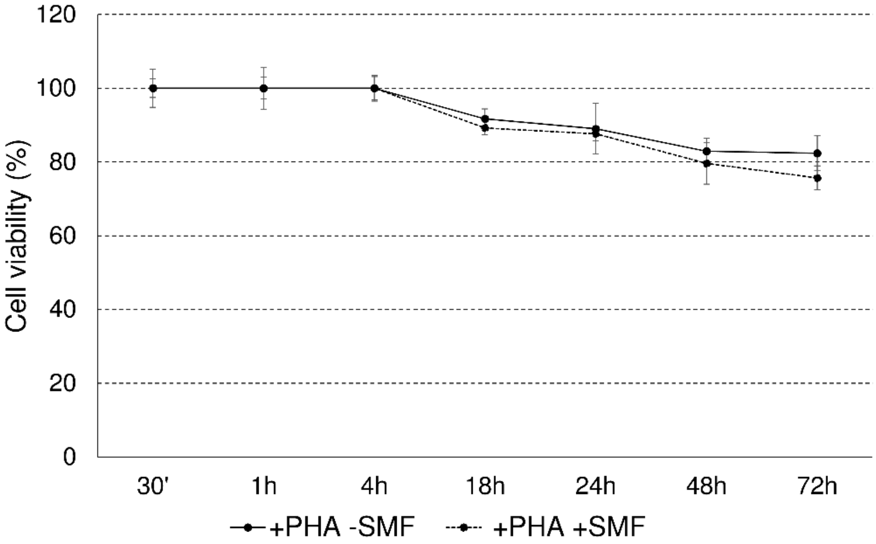

Figure 2 Supplementary material. Cell viability evaluated by MTT assay from 30 minutes to 72 h of PBLs supplemented with PHA (10µg/mL) in the presence and in the absence of 6 mT SMF. All values are referred to the value of control PBLs at 0 h (before the start of time course), taken as 100%. Each error bar represents the SE of five independent experiments, performed in duplicate.

Figure 3 Supplementary material shows the uptake experiment using PBLs exposed to 6 mT SMF for 24h before incubation with AgNPs..

The results demonstrate that i) also after 24h of exposure to SMF before incubation with AgNPs, the uptake occurs via lipid raft- and clathrin-mediated endocytosis; ii) the exposure to 6 mT SMF favours AgNPs internalization and the efficiency of uptake is high at 1 h of AgNPs-G incubation; iii) the ratio of internalization at 24h is lower that one measured at 72h, thus supporting the evidence that AgNPs uptake occurs via rafts, whose presence on the plasma membrane is, in turn, increased by SMF exposure.

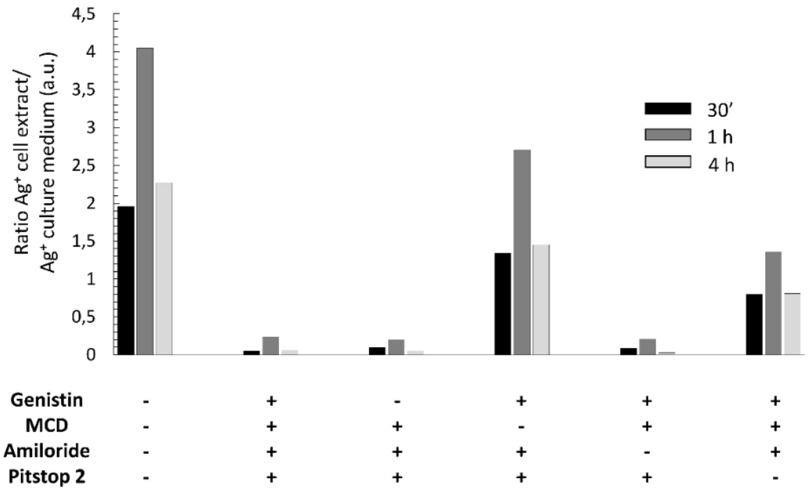

Figure 3 Supplementary material. ICP-OES analysis of the amount of AgNPs-G internalized by PBLs cells previously exposed to SMF for 24 h, treated with endocytic pathways selective inhibitors (Genistin, MCD, Amiloride, Pitstop 2) and further incubated with 2x103 AgNPs-G for 30 min, 1 h and 4 h. Data are reported as ratio between the Ag+ detected in cell extract and those detected in culture medium (absorbance in arbitrary unit, a.u.). (+) presence; (-) absence.
